# Outcomes of Allogeneic Stem Cell Transplant in Patients with Relapsed/Refractory Hodgkin Lymphoma

**DOI:** 10.3390/curroncol32020118

**Published:** 2025-02-18

**Authors:** Shiliang Ge, Kylie Lepic, Ravi Bhindi, Tobias Berg, Dina Khalaf, Brian Leber, Michael Radford, Irwin Walker, Gwynivere Davies, Alejandro Garcia-Horton

**Affiliations:** 1 Department of Medicine, McMaster University, Hamilton, ON L8N 3Z5, Canada; 2 Department of Oncology, McMaster University, Hamilton, ON L8V 5C2, Canada; 3 Michael G. DeGroote School of Medicine, McMaster University, Hamilton, ON L8P 1H6, Canada; 4 Centre for Discovery in Cancer Research (CDCR), McMaster University, Hamilton, ON L8S 4K1, Canada; 5 Escarpment Cancer Research Institute, Hamilton Health Sciences, Hamilton, ON L8L 0A4, Canada

**Keywords:** Hodgkin lymphoma, allogeneic stem cell transplant, relapse, refractory, PD-1 inhibitor

## Abstract

Background: The aim of this study was to evaluate real-world clinical outcomes and transplant-related complications of allogeneic stem cell transplantation (alloSCT) for Hodgkin lymphoma (HL). Methods: This was a single-centre, retrospective analysis of relapsed and refractory (R/R) HL patients who received an alloSCT between 1 January 2016 and 29 February 2024 in Hamilton, Ontario. The primary endpoint was overall survival (OS). The secondary endpoints were progression-free survival (PFS), non-relapse mortality (NRM), and graft-versus-host disease/relapse-free survival (GRFS). Results: Twenty-one patients were identified, with thirteen (62%) pre-treated with programmed death 1 (PD-1) blockade with either nivolumab or pembrolizumab. Seventeen (81%) patients underwent related haploidentical donor transplants, while four (19%) patients received a matched unrelated donor transplant. The 2-year OS and PFS rates were 79% (95% CI: 53–92%) and 63% (95% CI: 37–81%), respectively. Trends towards improved OS, PFS, NRM, and GRFS in PD-1-inhibitor-exposed patients were observed. All PD-1-inhibitor-exposed patients who were in complete remission proceeding to alloSCT remained alive at the last follow-up visit. Among the nine patients in partial remission at the time of alloSCT, three deaths were reported, with a 2-year OS of 61%. Conclusions: Our outcome data of a single-centre, heavily pre-treated cohort of Canadian patients confirm that alloSCT with post-transplant cyclophosphamide-based immunosuppression, which has been associated with improvements in PFS, remains a safe and feasible treatment option for patients with R/R HL in the era of checkpoint inhibitor use.

## 1. Introduction

The majority of patients with Hodgkin lymphoma (HL) are cured with frontline therapy, with a 5-year overall survival (OS) rate ranging from 96 to 99% in early-stage disease [1] and 56 to 89% in advanced-stage disease [2]. Advances in combination chemotherapy have also significantly improved the overall outcomes and cure rates of patients with HL. However, around 5–10% of HL patients remain refractory to frontline chemotherapy, with up to 30% of patients relapsing after achieving complete remission [3]. In this subset of patients, autologous stem cell transplant (ASCT) remains the standard of care given that second-line chemotherapy alone is associated with low long-term remission rates. However, the addition of novel agents to standard chemotherapy has resulted in improved response rates [4,5,6], with a recent study demonstrating improved efficacy in HL patients treated with nivolumab and doxorubicin, vinblastine, and dacarbazine (AVD) compared to HL patients treated with brentuximab vedotin (BV) and AVD [7]. Patients who progress after ASCT historically have had inferior clinical outcomes, with approximately 10% survival at 10 years and a median post-progression survival of only 2.1 years [8,9].

Patients with relapsed/refractory (R/R) disease pose a clinical challenge in terms of management. The available therapeutic options for relapsed disease post ASCT include antibody–drug conjugates such as BV and programmed death 1 (PD-1) blockade with nivolumab or pembrolizumab [3]. Generally, allogeneic stem cell transplant (alloSCT) is reserved as the last line of therapy in R/R HL patients [3]. The benefit of alloSCT in these patients remains controversial given mixed historical data underscoring the unclear benefit of alloSCT compared to sequential lines of therapy [10]. A large prospective study showed that while alloSCT in R/R HL reduced non-relapse mortality (NRM), high disease relapse rates remain a significant clinical challenge [11]. The introduction of BV and PD-1 inhibitors has also heralded new treatment paradigms and prompted debate on the implications of alloSCT in R/R HL patients.

However, the advent of reduced-intensity and non-myeloablative regimens has led to lower toxicity profiles and the inclusion for consideration for alloSCT of older and less fit patients who were previously not transplant candidates [12]. A retrospective analysis of 168 patients demonstrated that the use of reduced-intensity conditioning (RIC) protocols significantly reduced NRM in HL patients who received alloSCT [13]. Supportive care and transplant methods have improved, specifically with the development of post-transplant cyclophosphamide graft-versus-host disease (GVHD)-based prophylaxis for alternative donor transplantation [12,14]. A clinically relevant graft-versus-lymphoma effect has also been demonstrated [15], with retrospective studies highlighting the curative potential of alloSCT in HL [10,16,17]. Recently, the advantage of haploidentical transplantation over matched donor transplantation has been debated. A large EBMT study demonstrated favourable outcomes with haploidentical transplant, showing a 2-year OS rate of 72% [18]. Another retrospective study of 198 HL patients reported enhanced progression-free survival (PFS) in patients receiving transplants from haploidentical donors compared to HLA-matched donors [19]. However, more recent studies have suggested that haploidentical alloSCT may be associated with an increased risk of acute GVHD (aGVHD) and may portend worse OS compared to alloSCT from HLA-matched donors [20,21]. The current recommendations do not identify a preferred donor (matched related or unrelated or haploidentical), except for cord blood grafts, as long as a post-transplant cyclophosphamide (PTCY) approach is used [22]. Together, these limited retrospective data suggest that alloSCT continues to be a viable therapy for R/R HL patients who show a response to therapy pre-transplant.

There remains an unmet clinical need in terms of the available treatment options for HL patients who relapse after ASCT. Data on the treatment of R/R HL patients remain limited, and more real-world data are required to better define management, especially in the era of novel agents where rates of complete remission are higher than those for previous standard multi-agent chemotherapy regimens. Our study was a single-centre retrospective outcome review of alloSCT for R/R HL to better elucidate the factors influencing the overall success of alloSCT in this patient population, as well as potential transplant-related complications. We hypothesized that alloSCT would be a safe and feasible treatment option despite paradigm shifts in novel treatment options for R/R HL.

## 2. Materials and Methods

### 2.1. Study Design and Participants

This was a single-centre, retrospective, observational study of R/R HL patients who received an alloSCT between 1 January 2016 and 29 February 2024 at the Juravinski Cancer Centre in Hamilton, Ontario, Canada. Institutional ethics board approval was obtained. The eligibility criteria for inclusion were as follows: (1) aged 18 years or older; (2) received an alloSCT; (3) HL identified as an indication for alloSCT; (4) two or more lines of therapy prior to alloSCT; (5) alloSCT performed between 1 January 2016 and 29 February 2024; (6) treated at the Juravinski Cancer Centre. Patients who did not meet all the inclusion criteria were excluded.

### 2.2. Data Collection

Consecutive patients who met the eligibility criteria were identified using the local clinical database, which also reports to the Center for International Bone and Marrow Transplant Research (CIBMTR). Electronic medical records were used as information sources. Observational data were extracted and collated, including patient demographics, comorbidities, disease characteristics, previous treatment history, transplant indications, disease outcomes, and post-transplant complications.

### 2.3. Definition of Endpoints

The primary endpoint for this study was OS, which was defined as the time from alloSCT until death from any cause. Any patients alive at the time of last evaluation were censored. The secondary endpoints included PFS, NRM, and graft-vs-host relapse-free survival (GRFS). PFS was defined as the time from alloSCT until the first evidence of lymphoma relapse, disease progression, or death. Patients alive and without evidence of relapsed disease or disease progression at the time of last evaluation were censored. NRM was defined as the time from alloSCT to death without evidence of lymphoma relapse. GRFS was defined as the absence of grade III-IV aGVHD, chronic GVHD (cGVHD) on systemic treatment, relapsed disease, or death.

### 2.4. Statistical Analysis

Descriptive statistics were used to summarize patient and disease-related variables for the study cohort. The Kaplan–Meier method was used to estimate the OS, PFS, and GFRS. Cumulative incidence functions were used to estimate the NRM. A univariate analysis was used to identify clinically significant variables. Given the small sample size, a multivariate analysis was not pursued. All statistical analyses were performed using STATA v16 (StataCorp 2019).

## 3. Results

### 3.1. Patient and Transplant Characteristics

A total of 21 patients with R/R HL who underwent alloSCT were included in this study. The patient and transplant characteristics are summarized in Table 1. The cohort consisted of 11 males (52%) and 10 females (48%), with a median age of 36 years (range: 20–66 years) at the time of transplant. The median number of treatment lines prior to alloSCT was 4 (range: 3–6), with all patients having received an ASCT prior to alloSCT. Nineteen (90%) patients were exposed to BV prior to alloSCT. BV was used for R/R disease rather than up-front in this cohort. A total of 13 (62%) patients were exposed to a PD-1 inhibitor (either nivolumab or pembrolizumab) prior to receiving an alloSCT. Of those, 11 (52%) had also been exposed to BV. At the time of transplantation, 7 patients (36%) were in complete remission (CR) and 14 patients (67%) were in partial remission (PR), as determined by computed tomography (CT) or positron emission tomography (PET) scans.

Of the 21 patients, 17 (81%) underwent related haploidentical donor transplants, while 4 (19%) received a matched unrelated donor transplant. Four (19%) transplants involved a female donor and male recipient. In this study cohort, 20 (95%) patients received fludarabine (30 mg/m^2^ days −6 to −2), cyclophosphamide (14.5 mg/kg days −6 to −2), and 200 cGy total-body irradiation on day −1 as a conditioning regimen. Only one patient received RIC with fludarabine (30 mg/m^2^ days −6 to −2) and melphalan (140 mg/m^2^ on day −3). All haploidentical donor transplant recipients received post-transplant cyclophosphamide (50 mg/kg on days +3 and +4), tacrolimus, and mycophenolate mofetil (MMF), starting on day +5, for GVHD prophylaxis. Three patients (14%) received anti-thymocyte globulin (4.5 mg/kg divided across days −2, −1, and +1), calcineurin inhibitor, and MMF-based regimens.

One patient received two related haploidentical donor transplants. Following relapsed disease after her first alloSCT, she was treated with nivolumab, which was later switched to pembrolizumab given Steven Johnson Syndrome, followed by BV, bendamustine, and radiation therapy. The second alloSCT occurred 58 months after the initial alloSCT and was complicated by grade III aGVHD and cGVHD of the gastrointestinal and genitourinary tracts. The second alloSCT was censored from the study for the analysis and outcomes. At last follow-up, her disease continued to be in remission.

### 3.2. Overall and Progression-Free Survival

**Figure 1:**
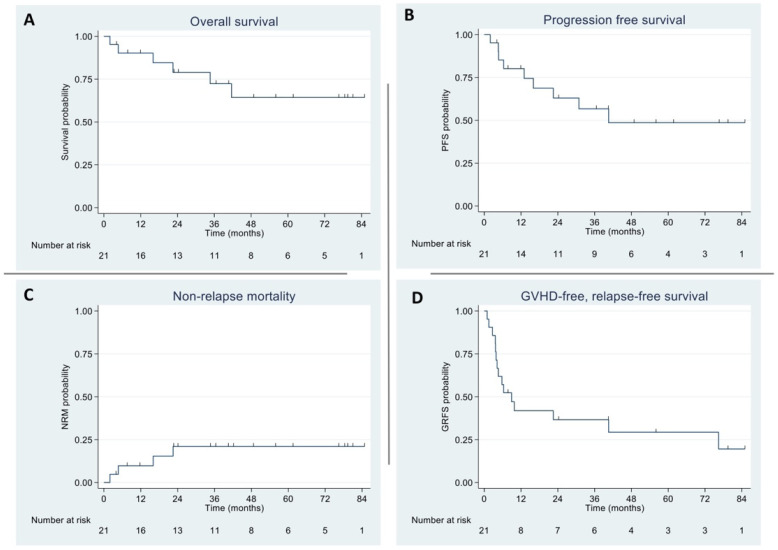
Outcomes of alloSCT in HL patients: (**A**) overall survival; (**B**) progression-free survival; (**C**) non-relapse mortality; (**D**) GVHD-free, relapse-free survival.

The 2-year OS rate was 79% (95% CI: 53–92%). The 2-year PFS rate was 63% (95% CI: 37–81%). The 4-year OS rate was reported to be 64% (95% CI: 36–83%), and the 4-year PFS rate was reported to be 49% (95% CI: 23–70%). This is summarized in Figure 1A,B. Two (33%) deaths were attributed to relapsed HL.

### 3.3. Non-Relapse Mortality and Graft-Versus-Host Disease/Relapse-Free Survival

The NRM rate was 10% (95% CI: 3–34%) at 1 year and 21% (95% CI: 8–47%) at 2 years. The causes of death were bacterial sepsis (n = 1), pneumonia (n = 1), fungal pneumonia (n = 1), and aGVHD (n = 1). The 2- and 4-year GRFS rates were 37% (95% CI: 17–57%) and 29% (95% CI: 11–51%), respectively. This is demonstrated in Figure 1C,D.

### 3.4. Transplant Response and Complications

The cumulative incidence of relapse in this patient cohort was 8.9% (95% CI: 3.7–21.4%). The transplant complications for this cohort are summarized in Table 2.

In our study cohort, cytomegalovirus (CMV) reactivation occurred in 14% of patients. Epstein–Barr virus (EBV) reactivation occurred in 10% of patients, with one patient diagnosed with post-transplant lymphoproliferative disorder. There were two cases of engraftment syndrome. Sixteen (76%) patients developed aGVHD, but only four of these cases were grade III-IV aGVHD. cGVHD developed in eight (38%) patients. A total of six (29%) patients died after receiving alloSCT (Table 2).

### 3.5. Outcomes by Prior PD-1 Inhibitor Exposure

**Figure 2:**
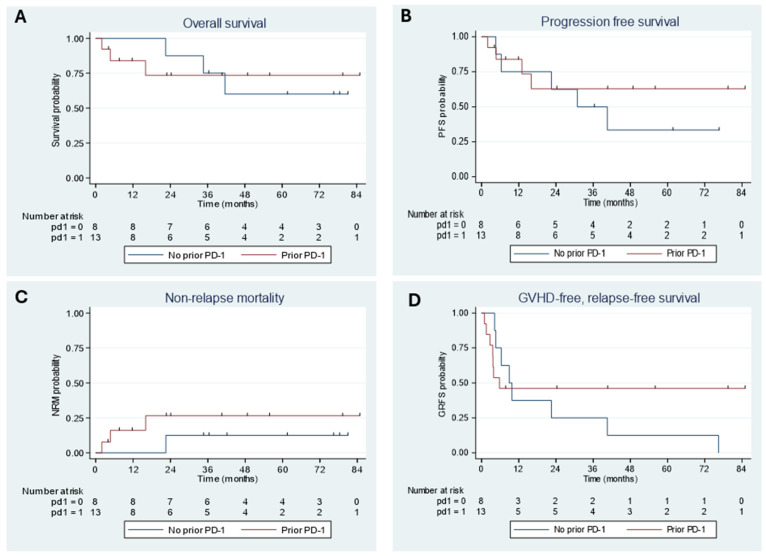
Outcomes of alloSCT in HL patients by PD-1 blockade exposure: (**A**) overall survival; (**B**) progression-free survival; (**C**) non-relapse mortality; (**D**) GVHD-free, relapse-free survival.

The outcomes by prior PD-1 inhibitor exposure are summarized in Figure 2. The 4-year OS rate in patients without PD-1 inhibitor exposure was determined to be 60% (95% CI: 20–85%). In comparison, the 4-year OS rate in patients with PD-1 inhibitor exposure was 73% (95% CI: 37–91%). These differences are only observed trends as our sample size is too small to draw definitive conclusions. The 4-year PFS rate in patients without PD-1 inhibitor exposure was 33% (95% CI: 6–66%), while the 4-year PFS rate in patients with PD-1 inhibitor exposure was 63% (95% CI: 28–85%). This also suggests a trend towards improved PFS with PD-1 inhibitor exposure. Similarly, trends were also noted towards improved outcomes in the 2-year GRFS rate in patients previously exposed to PD-1 inhibitors (46% in prior exposure vs. 25% in those without). The NRM rate was slightly higher at 2 years in patients with previous PD1 inhibitor exposure (27%) vs. no prior exposure (13%).

In patients who were exposed to PD-1 blockade, all patients who proceeded to alloSCT in CR were alive at the last study follow-up (n = 4), as compared to the nine patients transplanted in PR, among which three patients had died at the last study follow-up.

## 4. Discussion

The role and timing of alloSCT in the treatment of R/R HL remains controversial, and there is limited evidence to guide treatment decisions in this patient population. This was a single-centre retrospective observational study evaluating the outcomes of alloSCT in patients with R/R HL. Our findings indicate that alloSCT can lead to favourable long-term survival outcomes in R/R disease despite the challenges associated with advanced disease and previous treatment failures. This adds to the growing body of evidence highlighting the potential role of alloSCT in R/R HL.

Our outcome data of a single-centre, heavily pre-treated cohort of Canadian patients are comparable to those of other recently published cohorts, highlighting that in the era of post-transplant cyclophosphamide-containing conditioning, alloSCT is a safe and feasible treatment option for patients with R/R HL. The cumulative incidence of relapse, at 8.9%, is low, suggesting that alloSCT may provide effective disease control through the graft-versus-HL effect [13].

We reported 2-year OS and PFS rates of 79% and 63%, which are superior to previously reported data in a Canadian Blood and Marrow Transplant Group (CBMTG) review from 2010, which showed a 2-year OS rate of 35–60% and 2-year PFS rate of 25–30% in Canadian patients [23]. The improved outcomes seen with alloSCT in R/R HL over the last decade are likely related to improvements in supportive care, patient selection, and novel agents [24]. Our data are consistent with other recently published datasets. A large EBMT study including 240 HL patients confirmed favourable outcomes with haploidentical transplant, showing a 2-year OS rate of 72% and a 2-year PFS rate of 57% [18]. In that study, 77% of patients received RIC, and 33% received a myeloablative conditioning regimen (MAC) [18]. In comparison, 95% of the patients in our study received a non-myeloablative conditioning regimen (NMA) with Flu/Cy/TBI 200, and 5% of patients received RIC with Flu/Mel. This is in line with current practice preferences for adult patients with HL [22]. Another retrospective series of 98 patients in France and Belgium compared the outcomes of alloSCT with alternative donors (haploidentical, mismatched, unrelated, or cord blood), demonstrating an OS rate of 66–75% at 31 months [17]. In Gauthier et al.’s study, 60% of patients received RIC and 38% of patients received NMA. The majority of patients (79%) received four or fewer treatment lines prior to alloSCT, and 21% of patients were previously treated with BV [17]. This is similar to our study, where the median number of prior treatment lines was four. However, in our study, all patients were treated with either PD-1 blockade, BV, or both. The increasing prevalence in application of PD-1 blockade and antibody–drug conjugate therapies reflects the shifting landscape of available treatment options for R/R HL. The 4-year OS rate of 64% and the 4-year PFS rate of 49% described in our study are also promising. A retrospective study by Reyal et al. presented similar findings, with a 4-year OS rate of 65% (95% CI: 47–82%) and a 4-year PFS rate of 43% (95% CI: 23–64%) in patients with R/R HL who received a prior alloSCT [16]. Together, these findings suggest that alloSCT, specifically when using RIC and post-transplant cyclophosphamide, can be an effective treatment option for patients with R/R HL, providing durable remission in a significant proportion of patients.

Our study cohort was heavily pre-treated with a median of four treatment lines prior to receiving alloSCT. All 21 patients were treated with either BV or PD-1 inhibitors, with 11 patients having received both agents before proceeding with alloSCT. Chen et al. suggested that treatment with BV before RIC alloSCT may lead to prolonged PFS but reported no differences in OS. Their study described significantly improved 2-year PFS rates in patients who received BV (59.3%) compared to those who did not (26.1%) [25]. The introduction of immune checkpoint inhibitors also warrants a re-evaluation of our treatment paradigm in R/R disease. In the phase II KEYNOTE-087 study, half of the patients with R/R HL who achieved CR after treatment with pembrolizumab maintained a durable response for 4 or more years. The study also found that a second course of pembrolizumab for patients who experienced relapse from initial CR produced sustained responses, with an overall response rate (ORR) of 73.7% among the 19 patients [26]. These data have led some clinicians to reconsider the role of alloSCT in R/R HL patients, especially in those who have achieved CR after treatment with PD-1 inhibitors. While the use of nivolumab and pembrolizumab in R/R HL has yielded promising results with durable response rates [26,27,28], the curative potential of PD-1 blockade is not fully understood. A longer-term follow-up of these studies demonstrated that a majority of patients eventually progressed on therapy [29]. There also remains concern that prior immune modulation with PD-1 blockade may adversely influence alloSCT outcomes, primarily given their association with an increased incidence of more severe aGVHD or sinusoidal obstruction syndrome (SOS) [30]. One retrospective analysis reported 1-year cumulative incidences of grade III-IV aGVHD of 23% and SOS of 8% in patients pre-treated with PD-1 inhibitors prior to alloSCT [31]. Another retrospective study reported nivolumab-induced aGVHD occurring in 6 out of 20 HL patients relapsing post alloSCT, with two GVHD-associated deaths [32]. Moreover, disease relapse post alloSCT remains a challenge given the limited treatment options, and PD-1 blockade has been recommended for use in this setting [32].

However, a study by De Philippis et al. suggested that checkpoint inhibition before haploidentical alloSCT actually improved PFS in patients receiving haploidentical alloSCT with cyclophosphamide as a GVHD prophylaxis [33]. These studies raise questions on the utility and timing of alloSCT in R/R HL with PD-1 blockade, given that many of the patients pre-treated with PD-1 blockade are potential candidates for alloSCT. AlloSCT offers a potential curative option for R/R HL patients who achieve PR or CR [11,34], although it remains unclear whether or not PR or CR status confers improved OS and PFS. While some studies suggested improved OS and PFS with CR pre-treatment [18,24], Gauthier et al. did not cite any significant difference [17]. Our study cohort was heavily pre-treated, with 13 (62%) patients pre-treated with PD-1 inhibitors with nivolumab or pembrolizumab. Similarly to Koyun et al. and De Philippis et al., we found that there were trends towards improved OS and PFS in patients who were exposed to PD-1 inhibitors prior to alloSCT [24,33]. We also showed a trend towards excellent response, with no deaths in patients with prior PD-1 blockade exposure who proceeded to alloSCT in CR (2-year OS rate of 100%), compared to three deaths among patients transplanted in PR (2-year OS rate of 61%). However, given our limited sample size, only trends are reported, and no statistical significance can be confirmed or drawn from this study [34]. Overall, there remains a need for larger prospective trials to evaluate the use of PD-1 inhibitors and alloSCT, especially given that reducing long-term morbidity from therapy remains a significant consideration for young R/R HL patients.

Several limitations must be acknowledged. This study’s retrospective design may have introduced potential selection bias in the reporting outcomes, as patients who ultimately did not proceed to alloSCT were not evaluated. However, the comprehensive nature of our centre’s transplant database ensures that data from all consecutive transplant patients would have been captured and analysed. Furthermore, the small sample size and shorter follow-up time limits the generalizability of our findings and precludes evaluation of long-term outcomes. Given our small sample size, we were unable to identify significant factors that could delineate patients who would benefit more, or less, from alloSCT and improve patient selection for this therapy. Hence, we were also unable to complete a multivariate analysis. Future studies are necessary to validate our findings, identify prognostic factors through multivariate analysis, and better delineate the optimal timing of alloSCT in HL in the era of novel therapies.

## 5. Conclusions

The management of R/R HL remains a significant challenge, particularly in patients who have exhausted conventional treatment options including high-dose chemotherapy and ASCT. AlloSCT represents a viable treatment option for R/R patients, with significant implications for long-term survival. Our study overall shows excellent feasibility and confirms positive outcomes in heavily pre-treated R/R HL patients receiving alloSCT with post-transplant cyclophosphamide-based immunosuppression, highlighting the curative potential of this treatment modality. Continued investigations into patient selection criteria and the optimal timing of transplants, including an understanding of prior PD-1 blockade use, response, and washout strategies, are essential in enhancing the therapeutic efficacy of alloSCT in the R/R HL population.

## Figures and Tables

**Table 1:** Study cohort characteristics.

Characteristic		N (Total = 21)
Age, years, median (range)		36 (20–66)
Sex, n (%)	Male	11 (52%)
	Female	10 (48%)
HL subtype, n (%)	Classical (no subtype)	4 (19%)
	Nodular sclerosing	13 (62%)
	Mixed cellularity	4 (19%)
HCT-CI, n (%)	0	4 (19%)
	1–2	9 (43%)
	3–4	5 (24%)
	>5	2 (9%)
	Missing	1 (5%)
Karnofsky Performance Status, n (%)	≥90	14 (67%)
	<90	7 (33%)
Status at transplantation	CR	7 (36%)
	PR	14 (67%)
Number of prior treatment lines, median (range)	4 (3–6)	
Prior ASCT, n (%)	21 (100%)	
Prior BV exposure, n (%)	19 (90%)	
Prior PD1 inhibitor exposure, n (%)	13 (62%)	
Prior BV and PD1 inhibitor exposure, n (%)	11 (52%)	
Time from diagnosis to transplant, years, median (range)	3.1 (1.9–12.3)	
Donor type, n (%)	Related, haploidentical	17 (81%)
	Matched unrelated	4 (19%)
Stem cell source, n (%)	PBSC	21 (100%)
Female donor to male recipient, n (%)	4 (19%)	
Stem cell dose (CD34 × 10^6^ cells/recipient kg), median (range)	6.4 (3.9–11.8)	
Fresh vs. cryopreserved, n (%)	Fresh	19 (90%)
	Cryopreserved	2 (10%)
Conditioning regimen, n (%)	Flu/Cy/TBI 200	20 (95%)
	Flu/Mel	1 (5%)
GVHD prophylaxis, n (%)	PTCy/tacro/MMF	18 (86%)
	ATG/CNI/MMF	3 (14%)
CMV status, n (%)	R+/D+	4 (19%)
	R+/D−	1 (5%)
	R−/D−	11 (52%)
	R−/D+	5 (24%)
ABO mismatch, n (%)	None	9 (43%)
	Major	3 (14%)
	Minor	7 (33%)
	Missing	2 (10%)

Abbreviations: HL, Hodgkin lymphoma; HCT-CI, Hematopoietic Cell Transplantation-specific Comorbidity Index; ASCT, autologous stem cell transplant; GVHD, graft-versus-host disease; CMV, cytomegalovirus.

**Table 2:** Post-transplant complications.

Complication		N (Total = 21)
CMV reactivation, n (%)	No	17 (81%)
	Yes	3 (14%)
EBV reactivation, n (%)	No	19 (90%)
	Yes	2 (10%)
	PTLD	1 (5%)
BK virus infection, n (%)	No	20 (95%)
	Yes	1 (5%)
Engraftment syndrome, n (%)	No	19 (90%)
	Yes	2 (10%)
VOD/SOS, n (%)	1 (5%)	
aGVHD (all grades), n (%)	16 (76%)	
aGVHD, grade III-IV, n (%)	4 (19%)	
cGVHD, extensive, n (%)	8 (38%)	
Deaths, n (%)	6 (29%)	
Cause of death, n (%)	Bacterial sepsis	1 (17%)
	Pneumonia	1 (17%)
	Fungal pneumonia	1 (17%)
	aGVHD	1 (17%)
	Hodgkin lymphoma	2 (33%)

Abbreviations: CMV, cytomegalovirus; EBV, Epstein–Barr virus; VOD, veno-occlusive disease; SOS, sinusoidal obstruction syndrome; GVHD, graft-versus-host disease.

## Data Availability

Data available on request from the authors.
